# Deaths from Norovirus among the Elderly, England and Wales

**DOI:** 10.3201/eid1410.080188

**Published:** 2008-10

**Authors:** John P. Harris, W. John Edmunds, Richard Pebody, David W. Brown, Ben A. Lopman

**Affiliations:** Health Protection Agency, London, UK

**Keywords:** Mortality, norovirus, gastrointestinal diseases, research

## Abstract

The number of deaths in England and Wales associated with gastrointestinal pathogens, norovirus in particular, in persons >65 years was estimated for 2001–2006. Regression analysis was used to model monthly counts of gastrointestinal pathogens in fecal samples from infected patients against monthly counts of deaths from infectious and noninfectious intestinal diseases. Data came from the Office of National Statistics (death registrations from local registrars) and from the Health Protection Agency (laboratory results). Model results suggest that 20% (13.3%–26.8%) of deaths in persons >65 years of age caused by infectious intestinal disease other than *Clostridium difficile* were associated with norovirus infection in this period and that 13% (7.5%–18.5%) of deaths caused by noninfectious intestinal disease were associated with norovirus. An estimated 80 deaths each year in this age group may be associated with norovirus infection.

## Abstract 2

Norovirus may be associated with ≈80 deaths/year.

Estimating the number of deaths associated with infection is challenging. Deaths resulting from infectious diseases tend to be underreported on death certificates (*1*). Similarly, laboratory reports record the pathogens detected but rarely record long-term outcomes, such as death or other sequelae. Routine hospital admissions and discharge data only record deaths that occur in hospital, and coding may not always be complete or accurate, especially if diagnostic results are not available (*2*,*3*).

Norovirus is the most common cause of acute gastrointestinal infections and causes most reported outbreaks of gastrointestinal disease in England and Wales (*4*). Outbreaks occur more often during the winter months of October to March (*5*), but occasionally unexpectedly high activity can occur during the summer months (*6*). To date, no published data estimate the number of deaths from norovirus infections in the United Kingdom. We estimated the number of deaths associated with gastrointestinal pathogens by using previously reported methods (*1*,*2*,*7*–*9*) and, in particular, we estimated the seasonal contribution of norovirus to death in the elderly (>65 years of age). We also tested the hypothesis that the 2002–03 norovirus season, when a novel strain emerged (*10*), was associated with more pathogenicity than were other norovirus seasons.

## Methods

### Data Sources

#### Laboratory Reports

The Health Protection Agency collects data from laboratories around England and Wales on pathogens identified in fecal samples from infected patients with gastrointestinal symptoms (*11*). Samples come from persons in the community (taken by general practitioners), from persons involved in outbreaks (taken by Environmental Health Officers), and from hospitalized patients. Monthly counts (based on the date the specimen was taken) of positive specimens for January 2001–December 2006 were extracted for those >65 years of age. The organisms responsible for gastrointestinal diseases extracted for the analysis were *Salmonella* spp., *Shigella* spp., *Campylobacter* spp., *Escherichia coli* (not Shiga-toxin producing), enteric adenovirus, rotavirus, astrovirus, norovirus, *Cryptosporidium* spp., and *Giardia* spp. All other intestinal parasitic diseases were grouped together as other parasites. All bacterial pathogens were grouped by genera; e.g., all *Campylobacter* spp. were grouped together.

#### Mortality Statistics

The Office of National Statistics (ONS) compiles mortality statistics based on death registrations from local registrars in England and Wales. Cause-of-death information on death certificates is coded by ONS according to the International Classification of Diseases, 10th revision. Annually, ONS provides HPA with a file of all deaths that mention an infectious disease code. Deaths with a code for an infectious intestinal disease (ID), either as the underlying cause of death or a contributing cause of death, were extracted for 2001–2006 for persons >65 years of age (Table 1). We repeated the exercise for deaths that were considered to be caused by noninfectious ID. Deaths with any mention of *Clostridium difficile* were excluded from the analysis.

**Table 1:** International Classification of Diseases, 10th Revision, codes used for defining deaths from infectious and noninfectious causes, England and Wales, 2001–2006

Code	Diagnosis
A00	Cholera
A01	Typhoid and paratyphoid fevers
A02	Other *Salmonella* infections
A03	Shigellosis
A04	Other bacterial intestinal infections (excludes A047, *Clostridium difficile*)
A05	Other bacterial foodborne intoxications
A06	Amebiasis
A07	Other protozoal intestinal diseases
A08	Rotaviral enteritis
A09	Diarrhea and gastroenteritis of presumed infectious origin
A212*	Pulmonary tularemia
A213*	Gastrointestinal tularemia
B462*	Gastrointestinal mucormycosis
K22*	Other diseases of esophagus
K229	Disease of esophagus, unspecified
K29*	Gastritis and duodenitis
K299	Gastroduodenitis, unspecified
K31*	Other diseases of stomach and duodenum
K319	Disease of stomach and duodenum, unspecified
K521	Toxic gastroenteritis and colitis
K528	Other specified noninfective gastroenteritis and colitis
K529	Noninfective gastroenteritis and colitis, unspecified
K92*	Other diseases of digestive system
K929	Disease of digestive system, unspecified
T47*	Poison agents primarily affecting the gastrointestinal system
T478*	Poisoning by other agents primarily affecting the gastrointestinal system
T479*	Poisoning by agent primarily affecting the gastrointestinal system unspecified
Y53*	Agents primarily affecting the gastrointestinal system
Y538*	Other agents primarily affecting the gastrointestinal system
Y539*	Agent primarily affecting the gastrointestinal system, unspecified

*Although these codes were used in the search, none yielded any results for use in our dataset.

### Statistical Analyses

Because most gastrointestinal pathogens are highly seasonal, we estimated the number of gastrointestinal-related deaths by regressing monthly counts of laboratory reports on monthly counts of deaths. As we could not assume monthly counts of deaths to be normally distributed, simple linear regression models were inappropriate. We modeled monthly counts of deaths as a Poisson distribution, which has properties appropriate for analysis of count data. We used generalized linear regression models, which are used to extend simple linear regression to incorporate other distributions, to model monthly deaths as a Poisson-distributed outcome of laboratory reports of gastrointestinal pathogens.

Poisson regression also assumes that the data are not overdispersed (i.e., the variance is equal to the mean). Negative binomial models relax this assumption; we also considered negative binomial models, although they gave no qualitative differences in the results. To estimate the number of deaths that may be attributed to each pathogen, all models were fitted by using STATA 10.0 (*12*).

Our approach assumed a fixed proportion of laboratory reports for each organism to deaths over the period examined. The initial model included all laboratory reports for the extracted organisms as explanatory variables (Table 2). A constant term was included in all models to account for deaths not explained by the seasonal variation in laboratory reports. Because the number of deaths reported in each year (from both infectious and noninfectious ID) exhibited an upward trend during the study period, an independent term, consisting of year and month, was fitted to the model to account for this trend. In the initial full model, monthly deaths were modeled as a function of laboratory reports for each gastrointestinal pathogen (11 terms), the linear time variable, and a constant term (Table 2). Pathogens were removed if the coefficient was negative (because that was considered not biologically plausible) or if the variable was not significant in the model (p>0.05) to give the most parsimonious model. Model coefficients are on the natural scale (i.e., they represent directly how many deaths are associated with each laboratory report). The number of deaths in each month was estimated by multiplying the coefficient from the regression model for the pathogen by the number of monthly laboratory reports for that pathogen. Norovirus activity is highly seasonal; therefore, the number of deaths was also calculated with the year beginning in July and ending in June.

**Table 2:** Regression model results for deaths from infectious and noninfectious gastrointestinal disease in persons >65 years of age, England and Wales, 2001–2006

Pathogen	Initial full model			Final model	
	Coefficient*	p value†		Coefficient*	p value†
Infectious intestinal disease models					
Norovirus	0.0134	0.003		0.0174	<0.001
Astrovirus	–0.059	0.415		–	
*Shigella* spp.	0.103	0.528		–	
Rotavirus	–0.055	0.003		–	
*Campylobacter* spp.	–0.017	0.001		–	
*Escherichia coli*	0.067	0.612		–	
*Cryptosporidium* spp.	–0.151	0.122		–	
*Giardia* spp.	–0.165	0.209		–	
Other parasites	–0.012	0.890		–	
*Salmonella* spp.	–0.011	0.478		–	
Adenovirus	–0.341	0.266		–	
Time trend	1.437	<0.001		1.611	<0.001
Constant	23.11	<0.001		6.239	<0.001
Noninfectious intestinal disease models					
Norovirus	0.0134	0.011		0.0173	<0.001
Astrovirus	0.115	0.198		–	
*Shigella* spp.	0.240	0.221		–	
Rotavirus	–0.066	0.004		–	
*Campylobacter* spp.	0.001	0.903		–	
*E. coli*	–0.088	0.566		–	
*Cryptosporidium* spp.	–0.125	0.315		–	
*Giardia* spp.	–0.030	0.860		–	
Other parasites	–0.081	0.439		–	
*Salmonella* spp.	–0.039	0.050		–	
Adenovirus	0.0147	0.967		–	
Time trend	2.008	<0.001		2.488	<0.001
Constant	20.240	<0.001		11.135	<0.001

*The coefficient represents the number of deaths associated with each laboratory report for each pathogen. The constant (intercept term) indicates monthly deaths associated with other causes. †Wald test.

We carried out 2 additional analyses to test whether the 2002–03 season, when a novel norovirus strain emerged, was associated with increased pathogenicity. First, we tested for an interaction between the 2002–03 season and laboratory reports of norovirus. We looked for a significant difference in the relationship between laboratory reports of norovirus and deaths in 2002–03 season compared with other seasons (i.e., effect modification). A higher coefficient would indicate higher pathogenicity in this epidemic season. Secondly, we calculated a ratio of deaths to laboratory reports by dividing the number of deaths with any direct mention of viral gastroenteritis on the death certificate by the number of laboratory reports of norovirus in the corresponding year.

## Results

**Figure 1:**
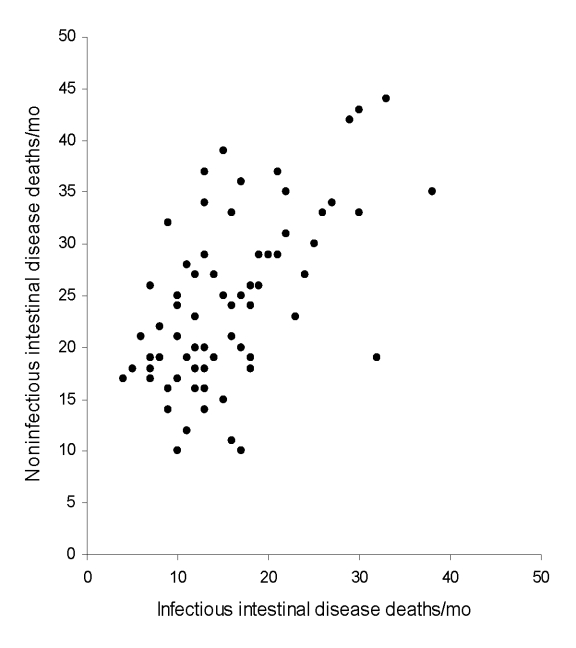
Correlation of monthly death reports of infectious and noninfectious intestinal disease, 2001–2006.

**Figure 2:**
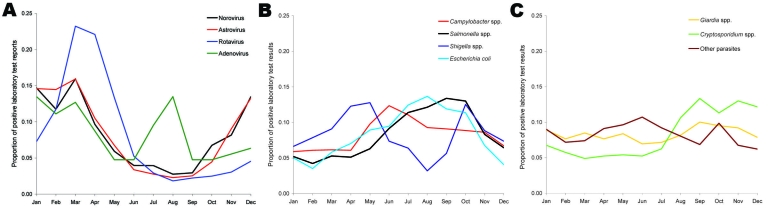
Seasonality of positive laboratory reports of viral (A), bacterial (B), and parasitic (C) pathogens, 2001–2006, persons >65 years of age.

During 2001–2006, a total of 1,136 deaths were recorded with any code for infectious ID and 1,736 for noninfectious ID (Figure 1). Infectious and noninfectious ID–associated deaths were correlated (R^2^ = 0.33, p = 0.10, Figure 1) and exhibited a wintertime seasonal pattern. Over the same period (2001–2006) in England and Wales, a total of 65,932 laboratory reports of the pathogens of interest were submitted for those >65 years of age. Summertime seasonality of the major bacterial pathogens and the wintertime seasonality of viral pathogens for this age group are illustrated in Figure 2, panels **A**–**C**.

Table 2 shows the comparisons of the best fitting models for infectious and noninfectious ID–associated deaths. Norovirus was the only pathogen significantly associated with monthly counts of infectious ID deaths (p<0.001). All other pathogens were removed from the model. In the noninfectious ID deaths model, astrovirus was also significant (p = 0.02). Because astrovirus infection is rare in the elderly (*4*) and shares a similar seasonality with norovirus, we decided to leave it out of the final model. A linear term, accounting for the general increasing trend in the number of deaths reported, significantly improved the model (p<0.001) and was included in the final estimation. Norovirus remained the only significant pathogen in the model. Therefore, in the final models the expected number of norovirus deaths was modeled as a Poisson distribution of laboratory reports of norovirus and a linear time variable. Some slight overdispersion was evident in the infectious ID deaths model, but fitting an alternative model to account for the overdispersion, negative binomial models, did not substantially alter the results, which suggests that Poisson regression is not inappropriate in this case.

**Figure 3:**
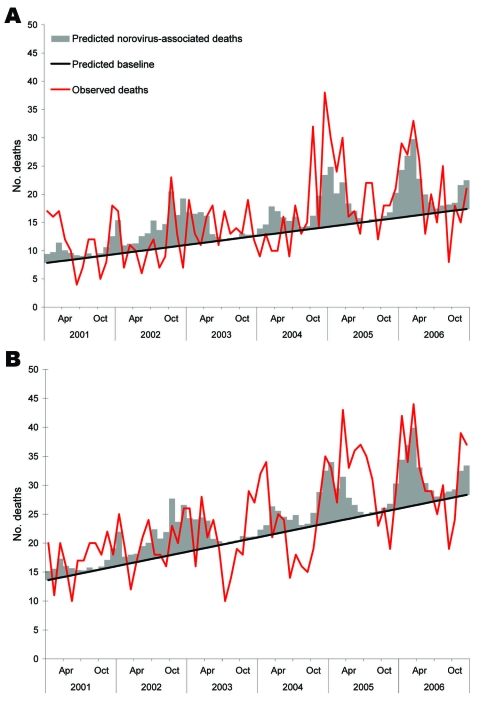
Observed and expected monthly deaths in persons >65 years of age from infectious intestinal diseases (A) and noninfectious intestinal diseases (B), derived from the most parsimonious models.

The model estimates that during 2001–2006, a total of 228 deaths from infectious ID were associated with norovirus infection, which represents 20% (13.3%–26.8%) of deaths from infectious ID in those >65 years of age; 225 (13% [7.5%–18.5%]) of deaths from noninfectious ID were associated with norovirus. Thus, the annual average number of deaths (January to December) from both infectious ID and noninfectious ID was 38; however, when looking at the period from July to June in each year, to account for the norovirus season, the average was ≈40 each season (Table 3). Figure 3, panels **A**, **B**, illustrates that models fit better to the deaths from infectious ID but still show some association with the deaths from noninfectious ID.

**Table 3:** Estimated number of deaths in each season (July to June) from the regression models, England and Wales, 2001–2006

Year	Predicted annual deaths, no. (95% confidence interval)		
	Infectious intestinal disease only	Noninfectious intestinal disease only	Infectious and noninfectious intestinal disease
2001–02	22.2 (14.7–29.7)	21.9 (12.7–31.2)	44.3 (32.4–56.2)
2002–03	59.5 (39.4–79.5 )	58.8 (34.0–83.7)	118.9 (86.9–150.8)
2003–04	18.4(12.2–24.6)	18.2 (10.5–25.9)	36.8 (26.9–46.7)
2004–05	51.4(34.1–68.8)	50.9 (29.4–72.4)	102.8 (75.2–130.5)
2005–06	51.8(34.3–69.3)	51.3 (29.6–72.9)	103.6 (75.7–131.4)
Total	203.3 (134.6–272.0)	201.2 (116.1–286.2)	406.5 (297.2–515.7)
Annual mean	40.7	40.2	81.3

**Figure 4:**
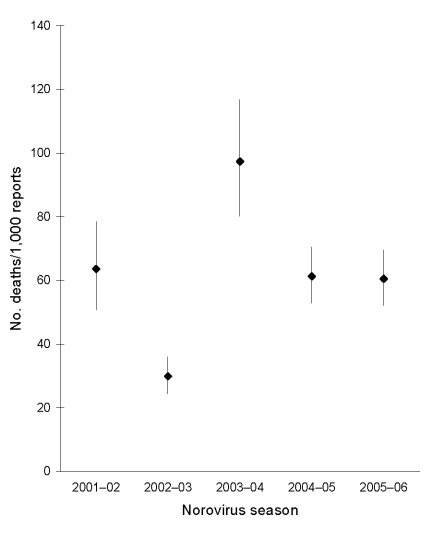
Ratio of viral gastroenteritis–associated death reports to norovirus laboratory reports, 5 seasons.

In years with high seasonal activity, numbers of norovirus-associated deaths were higher. The overall death/laboratory report ratio for 2001–2006 was 55/1,000 (95% confidence interval [CI] 51–60). The ratio did not increase in the years with greater numbers of deaths (Figure 4), and we found no evidence that the ratio was significantly higher during any of the study years. The 2002–03 season had the lowest death/laboratory report ratio. Including an interaction term in the infectious ID model between the epidemic 2002–03 season and laboratory reports of norovirus resulted in a negative-coefficient interaction term (likelihood ratio test p value = 0.002). This finding suggests a lower death/laboratory report ratio in the 2002–03 season, contrary to the hypothesis we were testing. The relative risk for death in the 2002–03 season compared with all other seasons was 0.81 (95% CI 0.69–0.96, p = 0.016).

In an analysis of recorded causes for all infectious ID deaths, viral gastroenteritis was specifically listed as an underlying cause for 13.4% of deaths (152/1,136). Table 4 shows the distribution of recorded underlying causes when viral gastroenteritis was mentioned as a contributory cause in 20% (227/1,136) of infectious ID deaths. For noninfectious ID deaths, diseases of the digestive system accounted for 52% (898/1,736) of the recorded underlying causes. Of these, 92% (829/898) were caused by unspecified noninfectious gastroenteritis and colitis.

**Table 4:** Recorded underlying causes of death in persons in whom viral infectious intestinal disease was a contributory factor

Underlying cause of death	%
Infectious intestinal disease (viral)	40.2
Circulatory system disorder	32.8
Respiratory system disorder	9.0
Neoplasm	4.9
Digestive system disorder	2.5
Nervous system disorder	2.5
Endocrine, nutritional, and metabolic disorders	2.5
Infectious intestinal disease (bacterial)	1.6
Mental/behavioral disorder	1.6
Musculoskeletal and connective tissue disorder	1.6
Genitourinary system disorder	0.8

## Discussion

Over the 6-year period of our study, the total number of deaths in persons >65 years of age that may be attributable to norovirus was 453 (228 from infectious ID and 225 from noninfectious ID). On average, this equates to ≈80 deaths each year attributable to norovirus infection. Norovirus was the only gastrointestinal pathogen that was consistently significant in the 2 regression models.

Of the recorded deaths from infectious ID, 13% had viral gastroenteritis listed as the underlying cause. For deaths from noninfectious ID, 48% had an underlying cause of unspecified noninfectious gastroenteritis and colitis. Because these are unspecified causes, and given their similar seasonality with infectious ID, many of these are likely to be infectious causes that were misclassified.

In years with higher norovirus activity, more deaths were associated with norovirus infection. However, we found no evidence of increased pathogenicity in years with higher recorded norovirus activity. The season when a new variant of the genotype II.4 virus emerged (2002–03) did not coincide with an increase in death/laboratory-report ratio. Indeed, the opposite was observed; fewer deaths as a proportion of positive laboratory reports were observed, the interaction term showed a negative coefficient for that season, and the relative risk for death during that season was lower than during other seasons.

One of the assumptions in our regression model was that laboratory reporting was consistent over the time of the study. Laboratory reporting processes did not change during the years of the study and are unlikely to have caused bias in this study. Testing and reporting behavior, however, may have changed over time. The number of specimens identified by PCR and ELISA increased from ≈70% to ≈90% in the study period. Thus, the decreased ratio of deaths/laboratory reports may have resulted from increased testing during the 2002–03 season rather than from the virus being less pathogenic during that year.

The modeling approach may underestimate the contribution of norovirus and other pathogens because the method estimates how much of the seasonal variation in death is associated with the seasonal variation in laboratory reports. Less-seasonal pathogens are less likely to show an association, and nonseasonal components (i.e., background levels) will not be attributed to a pathogen. Indeed, a substantial constant term in our models represented these unattributed deaths. The model for deaths from noninfectious ID did not appear to be as good a fit as the model for deaths from infectious ID. There was, in our opinion, enough evidence of a correlation between infectious and noninfectious ID to make a case for including this model.

This method has been used in the past for other pathogens (rotavirus, respiratory syncytial virus, pneumococcus, influenza virus) and unexplained deaths; when we used it in this study, we found an association between norovirus and death. Until our study, most reports of norovirus-associated deaths have been anecdotal (*13*). Although deaths associated with norovirus infection have been documented (*14*,*15*), these are usually singular reports of patients having died subsequent to infection with norovirus, rather than in-depth analysis of time trends of death.

In this study we attempted to go further and estimate the extent of death from norovirus. Norovirus is usually considered a mild, self-limiting disease, and most of those infected with the disease make a full recovery with no long-lasting effects. However, this study shows that part of the population, those >65 years of age, have a small risk of dying as a result of contracting norovirus. Rates of infection are higher within healthcare settings than in the community (*4*,*15*,*16*). Previous studies have shown that hospital patients who are involved in outbreaks of norovirus are ill longer than those who become infected in other settings (*15*). In England the proportion of the population >65 years of age is increasing. In years to come, this will be a substantial proportion of persons at risk, and deaths associated with this disease may well increase.

Noroviruses are known to evolve quickly. Emergence of new variants of the most commonly circulating strain can cause epidemic years in which more outbreaks occur and many more persons are infected. New variants are also associated with out-of-season activity, i.e., more outbreaks and infections than usual occurring in summer. When this happens, most of the population may be susceptible to infection. Our study suggests that when such epidemics occur, the number of norovirus-associated deaths increases as a result of the large number of persons infected rather than from increased virulence. Nevertheless, a measurable amount of death is associated with norovirus infection every year.
